# An analysis of effects of heterozygosity in dairy cattle for bovine tuberculosis resistance

**DOI:** 10.1111/age.12637

**Published:** 2018-01-24

**Authors:** S. Tsairidou, A. R. Allen, R. Pong‐Wong, S. H. McBride, D. M. Wright, O. Matika, C. M. Pooley, S. W. J. McDowell, E. J. Glass, R. A. Skuce, S. C. Bishop, J. A. Woolliams

**Affiliations:** ^1^ The Roslin Institute and R(D)SVS University of Edinburgh Edinburgh EH259RG UK; ^2^ Veterinary Sciences Division Agri‐Food and Biosciences Institute Belfast BT95PX UK; ^3^ School of Biological Sciences Queen's University of Belfast Belfast BT71NN UK

**Keywords:** disease resistance, dominance, genome‐wide association study, genomic selection

## Abstract

Genetic selection of cattle more resistant to bovine tuberculosis (bTB) may offer a complementary control strategy. Hypothesising underlying non‐additive genetic variation, we present an approach using genome‐wide high density markers to identify genomic loci with dominance effects on bTB resistance and to test previously published regions with heterozygote advantage in bTB. Our data comprised 1151 Holstein–Friesian cows from Northern Ireland, confirmed bTB cases and controls, genotyped with the 700K Illumina BeadChip. Genome‐wide markers were tested for associations between heterozygosity and bTB status using marker‐based relationships. Results were tested for robustness against genetic structure, and the genotypic frequencies of a significant locus were tested for departures from Hardy‐Weinberg equilibrium. Genomic regions identified in our study and in previous publications were tested for dominance effects. Genotypic effects were estimated through ASReml mixed models. A SNP (rs43032684) on chromosome 6 was significant at the chromosome‐wide level, explaining 1.7% of the phenotypic variance. In the controls, there were fewer heterozygotes for rs43032684 (*P *<* *0.01) with the genotypic values suggesting that heterozygosity confers a heterozygote disadvantage. The region surrounding rs43032684 had a significant dominance effect (*P *<* *0.01). SNP rs43032684 resides within a pseudogene with a parental gene involved in macrophage response to infection and within a copy‐number‐variation region previously associated with nematode resistance. No dominance effect was found for the region on chromosome 11, as indicated by a previous candidate region bTB study. These findings require further validation with large‐scale data.

## Introduction

Bovine tuberculosis (bTB) is an infectious zoonotic disease caused by *Mycobacterium bovis* and has devastating consequences for the UK cattle industry. Previous studies have demonstrated genetic variation in resistance to bTB (Bermingham *et al*. 2009; Brotherstone *et al*. 2010) and have shown the feasibility of genomic selection for bTB resistance using information from markers dispersed throughout the genome (Tsairidou *et al*. 2014). An alternative approach for selecting more resistant individuals is through identifying individual QTL that explain significant amounts of the genetic variance and exploiting this knowledge in marker‐assisted selection, which was examined for bTB resistance by Bermingham *et al*. (2014). Selection for disease resistance based on single QTL has previously been implemented successfully in aquaculture and livestock species: e.g. resistance to infectious pancreatic necrosis in Atlantic salmon (Houston *et al*. 2010) and resistance to *Escherichia coli* in pigs (Jørgensen *et al*. 2003; National Committee for Pig Production 2004).

Such studies typically assume that the QTL have additive effects. However, some of the phenotypic variation in disease resistance could also be explained by QTL with dominance effects. The increasing and decreasing expression of dominance is commonly assumed to underlie hybrid vigour and inbreeding depression respectively (Falconer & Mackay 1997; Oldenbroek 2007). Assuming a biallelic locus, over‐ or under‐dominance occurs when the heterozygote genotype is superior or inferior (respectively) to both homozygotes, and partial dominance occurs when the heterozygote is intermediate. For fitness, with over‐dominance, the selection is balancing and both variants would be expected to be maintained in a large population, whereas in other cases the population would be expected to move towards homozygosity for one of the alleles although it has reduced fitness. Over‐dominance is more likely than partial dominance to influence the decision on whether or not to fix a QTL with a favourable additive effect. Over‐dominance has been documented in disease traits, e.g. sickle cell anaemia in humans (Allison 1964) and crooked tail syndrome in Belgian Blue cattle (Fasquelle *et al*. 2009). Under‐dominance has also been documented, e.g. for the Rhesus blood group system in humans (Haldane 1941) and in establishing chromosomal rearrangements (White 1973) but, to our knowledge, not for resistance to infectious disease.

Therefore, the objective of this study was to detect non‐additive genetic variation associated with bTB resistance by searching for individual QTL exhibiting dominance effects. For this purpose, we used a genome‐wide association study (GWAS). This method has been widely used in human and animal studies to identify associations between genetic polymorphisms and traits through the use of large‐scale SNP data (Andersson 2009). However, the standard GWAS often fails to explore non‐additive genetic variation, and we present an adaptation directed towards this aim. A previous publication provided suggestive evidence of over‐dominance protecting heterozygotes from culling as a result of the bTB diagnostic SICCT test (Amos *et al*. 2013), which would be an important consideration shaping bTB eradication policy if it were confirmed. Therefore, an additional objective was to test these previous findings on a much larger dataset. Our findings are compared with results from standard genome scans, and the genomic regions identified are explored for candidate genes.

## Materials and methods

### Population

The dataset comprised 1151 Holstein‐Friesian cows of unknown pedigree from 165 herds in Northern Ireland; 592 of these were confirmed bTB cases and the remaining 559 were controls with multiple negative tuberculin test results. The available information included the cow's date of birth, testing dates and reasons for testing (e.g. routine surveillance testing and control tests within breakdowns); see Tsairidou *et al*. (2014) for further details. All cases were positive for the single intradermal comparative cervical test (SICCT) and had bTB lesions confirmed by carcass inspection, culture and molecular tests (Bermingham *et al*. 2014). Controls were age‐matched with cases and selected from herds with higher disease prevalence to increase their probability of exposure to the pathogen (Bishop 2012).

### Quality control

All individuals were genotyped with the 700K BovineHD Illumina BeadChip. Quality control was conducted within the genabel package (r/2.15.2) (Aulchenko *et al*. 2007b). Markers with less than 0.05% minor allele frequencies and less than 0.90% call rates were excluded. Animals were excluded when having less than 0.30% call rate, and one putative cow was discarded as it was a male (odds > 1000). This left 573 102 markers from 1150 individuals for calculating identity‐by‐state (IBS) relationships (see below). For the SNP of interest identified in the Results, the genotype call graph was examined for ambiguities, but none were found. The outcomes are given in Fig. S1, which shows that genotypic classes were found to form three distinct clusters, indicative of reliable genotyping.

### Genetic structure

The underlying genetic structure was examined using classical multidimensional scaling (CMDS) based upon pairwise distances between individuals calculated from the genome‐wide IBS information using the r/2.15.2 function ‘cmdscale’ (R Development Core Team 2008). CMDS revealed a secondary distinct cluster of 40 individuals, 39 of these from a single herd that included crossbreds, as had been observed previously (Bermingham *et al*. 2014). Therefore to address this, the analyses described below were repeated with and without this cluster.

### Heterozygote advantage GWAS

An adaptation of the GWAS was developed to identify non‐additive genetic variation. Firstly, genotypes were recoded as a binary heterozygosity indicator: so *d*_*ij*_ = 1 if individual *i* was a heterozygote at locus *j* and *d*_*ij*_ **= 0 otherwise. The ‘polygenic’ function of genabel was used to obtain residuals under a polygenic model (Aulchenko *et al*. 2007a,b) for use with the ‘mmscore’ function to test for associations between the *d*_*ij*_ and the bTB phenotypes. The polygenic model was:

(Model 1) y=m1+Xb+a+e,

where *y* is the binary bTB status (0: control; 1: case); *m* is the overall mean and **1** a vector of 1's; **b** is the vector of fixed effects with incidence matrix **X**, comprising the age of the individual as a covariate, year of testing, season of testing, the reason for testing and the two principal components calculated from the CMDS analysis on the genomic relationship matrix **G** (where data from the secondary cluster was included); **a** is the vector of polygenic breeding values with **a** assumed to follow a multivariate normal distribution, **a**~MVN (0, *σ*_a_^2^ **G**), where *σ*_a_^2^ is the additive genetic variance; and **e** is the residual error, assumed to be distributed MVN (0, *σ*_e_^2^ **I**).

The model was fitted within genabel, which calculates **G** following Amin *et al*. (2007):

$$g_{\mathrm{ij}}=n^{-1}\sum_{k=1}^{n}\left(x_{\mathrm{ik}}-2p_{k}\right)\left(x_{\mathrm{jk}}-2p_{k}\right)/\left[2p_{k}\left(1-p_{k}\right)\right]$$

$$g_{\mathrm{ii}}=1+n^{-1}\sum_{k=1}^{n}\left(H_{E,k}-H_{\mathrm{ik}}\right)/H_{E,k},$$

where *g*_*ij*_ is the genomic relationship between animals *i* and *j*;* n* is the number of loci used for estimating relationships; *x*_*ik*_ is the count of alternative alleles (0, 1 or 2) of individual *i* at SNP locus *k,* coded as 0, 1 and 2, where the reference allele is arbitrarily chosen; *p*_*k*_ is the frequency of the reference allele in the data; *H*_*E,k*_ is the expected heterozygosity at locus *k* assuming Hardy‐Weinberg equilibrium (HWE); and *H*_*ik*_ is the observed heterozygosity for animal *i* (0 or 1).

The model applied to each locus *k* by the ‘mmscore’ function was:

(Model 2) $$e=1m+b_{k}d_{k}+e_{k}^{*}$$

where *m* is the overall mean with **1** a vector of 1's; **d**_*k*_ is the vector of heterozygosity indicators for locus *k* and *b*_*k*_ is the associated regression coefficient; and **e**_k_* are residuals assumed to be distributed MVN (0, ν_e_^2^ **I**). To provide a check on consistency with the additive screen published by Bermingham *et al*. (2014), **d**_*k*_ was replaced by **x**_***k***_ in Model (Model 2), where **x**_*k*_ is the vector of counts for locus *k* described above.

A detailed analysis of departures from HWE was carried out for significant SNPs. The models and results are presented in Appendix S1.

### Significance thresholds applied

The genome‐wide degree of inflation (*λ*) for Model (Model 2) using the heterozygosity indicators **d**_*k*_ was 1.00 (SE = 1.02 × 10^−5^), indicating no serious inflation due to hidden structure in the data, although accounted for in significances quoted. Bonferroni corrections were applied to obtain genome‐ and chromosome‐wide significance thresholds, derived as −log_10_(0.05/*n*) and −log_10_(0.05/*n*_*c*_) respectively (where *n*_*c*_ is the number of SNPs on chromosome *c*). Particular attention was given to regions identified by Driscoll *et al*. (2011) and associated with a protective effect against reacting to the bTB diagnostic SICCT test (Amos *et al*. 2013), namely microsatellites *INRA111* (BTA11: 40 311 694–40 311 817 bp) and *BMS2753* (BTA9: 76 800 661–76 800 769 bp). Here, the 20 SNPs on each side of the two microsatellites were assessed using a significance threshold of −log_10_(0.05/80) = 3.2.

### Regional and genomic additive and dominance effects

The significance of the SNPs identified using Model (Model 2) and those flanking the microsatellites identified by Driscoll *et al*. (2011) was further tested using mixed linear models with both genome‐wide and regional additive and dominance effects fitted as random effects. The regional effects were defined by windows of 40 SNPs, 20 on each side of the locus of interest. The full model was:

y=m1+Xβ+a+d+α+δ+e,

where **y** is the binary bTB status (0: control, 1: case); *m* is the overall mean; **β** is the vector of fixed effects as described in (1) with incidence matrix **X**;** a** is the vector of additive polygenic effects with **a**~MVN(0, *σ*_a_^2^ **G**); **d** is the vector of polygenic dominance effects with **d**~MVN(0, *σ*_d_^2^ **D**); **α** is the vector of regional additive effects with **α**~MVN(0, v_a_^2^ **G**_**R**_), **δ** is the vector of regional dominance effects with **δ**~MVN**(0,** v_d_^2^ **D**_**R**_); and **e** is the vector of residual errors with **e** ~ MVN (0, **I** *σ*_e_^2^). The incidence matrices for all the random effects was **I** in this data. The (co)variance matrix **G** was as described above for Model (Model 1), and **G**_**R**_ was obtained using the same procedure using only the 40 SNPs defining the window. The (co)variance matrices of dominance effects **D** and **D**_**R**_ were calculated as:

$$d_{\mathrm{ij}}=m^{-1}\sum_{k=1}^{m}\left(z_{\mathrm{ik}}-2p_{k}q_{k}\right)\left(z_{\mathrm{jk}}-2p_{k}q_{k}\right)/{\left(2p_{k}q_{k}\right)}^{2},$$

where *d*_*ij*_ is the genomic relationship between individuals *i* and *j*;* m* is the number of SNPs included, *m *= *n* for **D** and 40 for **D**_**R**_; *z*_*ik*_ is the heterozygosity indicator for locus *k* of individual *i*;* p*_*k*_ is the frequency of the reference allele of locus *k* in the data; and *q*_*k*_ is frequency of the alternative allele. Likelihood ratio tests were conducted to identify significant regional effects with the genome‐wide terms **a** and **d** assumed to be nuisance factors and included in all models. In addition, alternative models were tested for the region containing the SNP with heterozygote disadvantage after excluding this SNP from the calculation of the local matrices.

## Results

### Additive GWAS model

Results from the standard GWAS are shown in Appendix S2. The SNPs identified in the present study were consistent with findings previously published by Bermingham *et al*. (2014) using the same data but different statistical procedures.

### GWAS for heterozygote advantage

**Figure 1:**
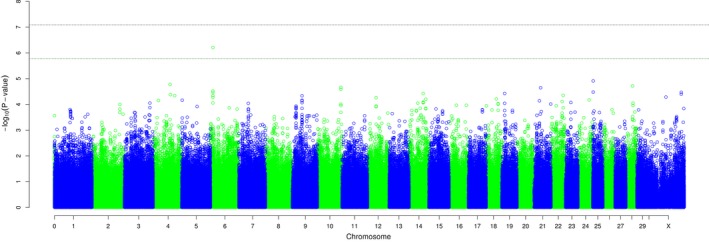
Heterozygote disadvantage GWAS Manhattan plot showing SNP associations with the bTB status. The green line represents the chromosome‐wide threshold for BTA6, and the black line represents the genome‐wide threshold.

The GWAS for heterozygote advantage identified a SNP on BTA6 (rs43032684) which was significant at the chromosome‐wide level with −log_10_ *P* = 6.21 (Table 1, Fig. 1). The rs43032684 locus on BTA6 was found to be consistently significant in both sample sets including and excluding the secondary cluster identified by CMDS (Table 1). The second most significant SNP identified, on BTA25 (rs109960101), did not reach chromosome‐wide significance (−log_10_ *P* = 4.91).

**Table 1:** Association *P*‐values for the SNP on BTA6 identified in the GWAS for heterozygote advantage on all the animals and after removing the animals in the minor cluster identified through classical multidimensional scaling (CMDS), with Bonferroni‐corrected significance thresholds

Analysis	rs43032684 –log_10_(*P*‐value)
All animals	6.21
After removel of CMDS‐identified cluster	6.09
Genome‐wide threshold	7.09
Chromosome‐wide threshold	5.78

None of the 80 SNP loci in the two regions identified by Driscoll *et al*. (2011) and Amos *et al*. (2013) achieved the lower statistical significance threshold. The most significant SNP locus on chromosomes BTA9 and BTA11 was rs110974556 on BTA9 with chromosomal position 40 944 275 bp, which is ~36 Mb away from the published microsatellite *BMS2753*, but this did not achieve chromosomal significance.

### Genotypic frequencies and deviations from HWE

The genotypic frequencies for rs43032684 on BTA6 are shown in Table 2. Departure from HWE was tested as described in Appendix S1, and maximum likelihood estimates for *p*_*A*_ and *α* for each line are shown in Table S2 for the four models tested. Likelihood ratio tests showed that the full model, with both *p*_*A*_ and *α* differing between lines, was a significantly better fit than any reduced model. Controls had a greater frequency of allele A (*p*_*A*_ = 0.728 cf. *p'*_*A*_ = 0.658; *P* < 0.01) and comparatively fewer heterozygotes, even after accounting for differences in *p*_*A*_ (*α* = 0.24 cf. *α*' = −0.02; *P* < 0.001) (Fig. S4). The cases showed no significant departure from HWE (*α*' = 0), whereas the controls showed excess homozygosity compared to HWE (*χ* ^2^ = 18.274, 1 d.f.; *P* < 0.001).

**Table 2:** Genotypic frequencies for rs43032684 on BTA6 for the cases and the controls

rs43032684	Cases	Controls	Fraction of total
A/A	0.21	0.25	0.46
A/G	0.22	0.13	0.35
G/G	0.05	0.05	0.11
*n*	0.48	0.44	–
Missing	0.03	0.05	0.08

### Regional and genomic additive and dominance variance

The region containing rs43032684, with apparent heterozygote disadvantage, contributed significant dominance variance (*P *<* *0.01) (Table 3). The dominance variance associated with this region remained significant after removing rs43032684 (also *P *<* *0.01), suggesting that the result was not due to SNP‐specific genotyping errors. No dominance variance was detected for either of the regions of microsatellites *INRA111* and *BMS2753* (Table 3). For the region containing *INRA111,* no additive genetic variance was detected. For *BMS2753*, Driscoll *et al*. (2011) had reported an unspecified genotype effect, and a small additive variance component (*P *=* *0.05) was detected in this region.

**Table 3:** Likelihood ratio test (LRT) for dominance and additive effects for the regions of interest. The full models contain the additive genomic matrix, the dominance genomic matrix, the local (40‐SNP) additive and the local dominance matrices; the reduced models are without the local dominance matrix. The alternative full model for the SNP with heterozygote disadvantage is for the same region but without the SNP of interest in the calculation of the local matrices

Model	BTA	Marker	Region	LogL	LRT	*P*‐value	d.f.
Full (1)	6	rs43032684	10 178 841–10 307 829	218.808			
Reduced (1)	6	rs43032684	10 178 841*–*10 307 829	213.603	10.410	<0.01	1
Full (2)	6	rs43032684	10 178 841–10 307 829	218.214			
Reduced (2)	6	rs43032684	10 178 841–10 307 829	213.570	9.288		1
Full	9	*BMS2753*	76 744 316–76 878 698	214.024			
Reduced	9	*BMS2753*	76 744 316–76 878 698	214.024			
Full	11	*INRA111*	40 196 811–40 503 568	211.732			
Reduced	11	*INRA111*	40 196 811–40 503 568	211.732			

LogL, log‐likelihood of the corresponding model; LRT = 2[LogL(full Model) – LogL(reduced Model)].

### Region exploration

The SNP identified through the heterozygote disadvantage GWAS (chromosomal position on BTA6: 10 245 091 bp) was found to be adjacent to several candidate genes including the *translocation associated membrane protein 1‐like 1* gene (*TRAM1L1*, chromosomal position on BTA6: 9 455 206–9 456 342 bp). A closer look at the surrounding region revealed that the SNP resides within two partly overlapping copy number variation (CNV) regions which were previously associated with resistance to gastrointestinal nematodes in cattle (Ensembl, UMD 3.1; BTA6: 9 524 225–10 295 238 bp and 9 914 188–10 346 486 bp) (Hou *et al*. 2011, 2012).

Furthermore, the SNP showing heterozygote disadvantage has two alleles (G/A) and resides within the *peroxiredoxin‐6 pseudogene* (*LOC784039*, BTA6: 10 223 720–10 246 027 bp, transcript length: 840 bps). The pseudogene contains two exons, and the SNP resides downstream of exon 1. Pseudogenes are homologues of functional parental genes, from which they have been derived through reverse mRNA transcription (processed pseudogenes) or through gene duplication (non‐processed pseudogenes) (Gerstein & Zheng 2006). In Ensembl, blasting the sequence of *peroxiredoxin‐6‐like pseudogene* against that of the *peroxiredoxin‐6* gene (BTA16) confirmed that *peroxiredoxin‐6* is the parental gene of *peroxiredoxin‐6‐like pseudogene* and that the latter is likely to be a processed pseudogene, as the parental gene resides on a different chromosome (Vanin 1985; Hirotsune *et al*. 2003; Kandouz *et al*. 2004; Bischof *et al*. 2006).

## Discussion

In this study we examined the hypothesis of a heterozygote (dis)advantage and a role for dominance in genetic resistance to bTB, which was proposed by Amos *et al*. (2013), and used a case–control population to test published candidate regions and other regions using high‐density SNP data. No evidence was found, neither a SNP effect nor dominance variance, to support the region previously published by Amos *et al*. (2013), which had been interpreted as showing a heterozygote advantage for resistance. In contrast, the findings of this study suggest that a region on BTA6 (rs43032684) may show dominance variance in bTB resistance, the SNP analysis suggesting a heterozygote disadvantage with heterozygotes more likely to be diseased. However, this association did not achieve genome‐wide significance and so emphasises a need for further validation. The additive effects identified through standard GWAS were consistent with findings previously reported by Bermingham *et al*. (2014) using the same data, although the two studies used different quality control and statistical procedures.

The putative heterozygote advantage reported by Amos *et al*. (2013) concerned a candidate microsatellite (*INRA111*), previously published by Driscoll *et al*. (2011) in a small mixed breed population. Two approaches to validation were adopted: one analogous to classical GWAS testing for associations with individual SNP loci within the candidate region and the second using regional variance components, which more closely models the haplotype structure of the regions and hence may be more appropriate for validating microsatellites, which were not included in the data. The lack of support for these candidates was despite the targeted testing procedures and strengthens the likelihood that the previous results were false positives. It is possible that the associations described by Amos *et al*. (2013) are not common to all breeds; however, the Holstein–Friesian gene pool studied here comprised a substantial proportion of the population investigated by Amos *et al*. (2013).

The region of the rs43032684 SNP on BTA6 was shown to harbour dominance variance in this study, but in contrast to the hypothesis of Driscoll *et al*. (2011) and Amos *et al*. (2013), the SNP identified appeared to show heterozygote disadvantage where by heterozygotes appeared to be over‐represented in cases compared to controls. The initial analysis, regressing the phenotype on the binary heterozygosity indicator, identified regions in which heterozygosity differs between cases and controls, but differences in heterozygosity can arise from changes in allele frequency (i.e. an additive effect). However, the analysis of genotype frequencies showed that the differences observed were due primarily to differences in the deviation from HWE between the cases and controls, which in effect, is consistent with some degree of heterozygote (dis)advantage. Nevertheless, the value of *α* was large and positive in the controls, and it would be expected that the controls would exhibit a degree of departure from HWE that is closer to the whole population than to the cases. This is because the prevalence of bTB is low, so the selection intensity in sampling the controls is smaller than that in sampling the cases. In the absence of selection, the value of *α* for random mating of female and male parents is expected to be negative but small (Robertson 1961) of the order of the rate of inbreeding, as in the cases, although the mating sub‐populations (such as farms) would tend to shift *α* towards positive values but to the extent observed for rs43032684. A random sample of 1500 SNP loci from chromosomes showing no evidence of associations with bTB resistance had a mean *α* = 0.0057 (SE = 0.0014) in the cases and *α* = 0.005 (SE = 0.0014) in controls and a mean difference between cases and controls of 0.0007 (SE = 0.0018). Therefore, the evidence presented for this region requires interpretation with caution and undoubtedly requires further validation.

The rs43032684 SNP resides within a pseudogene, but pseudogenes may retain some tissue‐specific and/or state‐specific functionality (Hirotsune *et al*. 2003; Kandouz *et al*. 2004). The parental gene product is peroxiredoxin‐6, an enzyme localised in lysosomes and active in lipid degradation pathways in various types of cells including alveolar macrophages (Sorokina *et al*. 2009; Chatterjee *et al*. 2011). Mycobacteria over‐ride the immune response by releasing lipid mediator molecules within the macrophages, which accumulate in the membrane of the phagosome, prohibiting its fusion with the lysosome, which would result in destroying the mycobacteria (Anes *et al*. 2003; Koul *et al*. 2004; Raman *et al*. 2010), therefore impairing clearance of infection (Gammack *et al*. 2004; Raman *et al*. 2010). Despite this apparent functional relevance, expression in monocytes could not be confirmed using PCR (K. Jensen, personal communication, January 18, 2013).

## Conclusions

We present here a genome‐wide association method for capturing non‐additive genetic variation, which allows for the identification of associations that are not detected via standard GWAS. In our study, we found no evidence for a region previously reported to have heterozygote advantage that would hinder culling of bTB infected cattle in the current eradication regime. In contrast, our methods identified a novel candidate region on BTA6 associated with bTB resistance, where locus heterozygosity was linked to increased susceptibility to bTB in cattle, i.e. a heterozygote disadvantage. The SNP was located in a pseudogene within a CNV region associated with nematode resistance in cattle; however, despite the apparent functional relevance, functionality was unable to be confirmed. Further studies are required to confirm these findings and validate the QTL on large scale data.

## Supporting information


**Figure S1** Genotype calls scoring graph for sequencing quality control for rs43032684, where the samples are displayed in three distinct shaded areas based on their genotype calls.

**Appendix S1** Departures from Hardy‐Weinberg equilibrium.
**Table S1** Estimates for rs43032684 on BTA6 of the frequency of allele A (*p*A), and the degree of departure from Hardy‐Weinberg equilibrium (α) for cases and controls with different constraints.
**Figure S2** Contour plots for the cases and controls showing departures from HWE.

**Appendix S2** Standard GWAS.
**Table S2** Chromosome‐wide significant SNPs identified from standard GWAS and corresponding *P*‐values.
**Figure S3** Manhattan plot from standard GWAS showing significance of SNP associations based on their *P*‐values.
**Figure S4** Q‐Q plot showing observed compared to expected x2 values under the null hypothesis of no association.

## References

[age12637-bib-0001] Allison A.C. (1964) Polymorphism and natural selection in human populations. Cold Spring Harbor Symposium on Quantitative Biology 29, 137–49. 10.1101/sqb.1964.029.01.018 14278460

[age12637-bib-0500] Amin, N. , van Duijn, C. M. and Aulchenko, Y. S. (2007) A genomic background based method for association analysis in related individuals. Plos One 2, e1274. 18060068 10.1371/journal.pone.0001274 PMC2093991

[age12637-bib-0002] Amos W. , Brooks‐Pollock E. , Blackwell R. , Driscoll E. , Nelson‐Flower M. & Conlan A.J.K. (2013) Genetic predisposition to pass the standard SICCT test for bovine tuberculosis in British cattle. PLoS One 8, e58245. 23554880 10.1371/journal.pone.0058245 PMC3605902

[age12637-bib-0003] Andersson L. (2009) Genome‐wide association analysis in domestic animals: a powerful approach for genetic dissection of trait loci. Genetica 136, 341–9. 18704695 10.1007/s10709-008-9312-4

[age12637-bib-0004] Anes E. , Kuhnel M.P. , Bos E. , Moniz‐Pereira J. , Habermann A. & Griffiths G. (2003) Selected lipids activate phagosome actin assembly and maturation resulting in killing of pathogenic mycobacteria. Nature Cell Biology 5, 793–802. 12942085 10.1038/ncb1036

[age12637-bib-0005] Aulchenko Y.S. , De Koning D.J. & Haley C. (2007a) Genomewide rapid association using mixed model and regression: a fast and simple method for genomewide pedigree‐based quantitative trait loci association analysis. Genetics 177, 577–85. 17660554 10.1534/genetics.107.075614 PMC2013682

[age12637-bib-0006] Aulchenko Y.S. , Ripke S. , Isaacs A. & Van Duijn C.M. (2007b) genabel: an r library for genome‐wide association analysis. Bioinformatics 23, 1294–6. 17384015 10.1093/bioinformatics/btm108

[age12637-bib-0007] Bermingham M.L. , More S.J. , Good M. , Cromie A.R. , Higgins I.M. & Brotherstone S. (2009) Genetics of tuberculosis in Irish Holstein‐Friesian dairy herds. Journal of Dairy Science 92, 3447–56. 19528623 10.3168/jds.2008-1848

[age12637-bib-0008] Bermingham M.L. , Bishop S.C. , Woolliams J.A. *et al* (2014) Genome‐wide association study identifies novel loci associated with resistance to bovine tuberculosis. Heredity 112, 543–51. 24496092 10.1038/hdy.2013.137 PMC3998787

[age12637-bib-0009] Bischof J.M. , Chiang A.P. , Scheetz T.E. , Stone E.M. , Casavant T.L. , Sheffield V.C. & Braun T.A. (2006) Genome‐wide identification of pseudogenes capable of disease‐causing gene conversion. Human Mutation 27, 545–52. 16671097 10.1002/humu.20335

[age12637-bib-0010] Bishop S.C. (2012) A consideration of resistance and tolerance for ruminant nematode infections. Frontier Genetics 3, 168. 10.3389/fgene.2012.00168 PMC3522420 23248638

[age12637-bib-0011] Brotherstone S. , White I.M.S. , Coffey M.P. , Downs S.H. , Mitchell A.P. & Clifton‐Hadley R.S. (2010) Evidence of genetic resistance of cattle to infection with *Mycobacterium bovis* . Journal of Dairy Science 93, 1234–42. 20172243 10.3168/jds.2009-2609

[age12637-bib-0012] Chatterjee S. , Feinstein S.I. , Dodia C. , Sorokina E. , Lien Y.‐C. , Nguyen S. , Debolt K. , Speicher D. & Fisher A.B. (2011) Peroxiredoxin 6 phosphorylation and subsequent phospholipase A2 activity are required for agonist‐mediated activation of NADPH oxidase in mouse pulmonary microvascular endothelium and alveolar macrophages. Journal of Biological Chemistry 286, 11696–706. 21262967 10.1074/jbc.M110.206623 PMC3064221

[age12637-bib-0013] Driscoll E.E. , Hoffman J.I. , Green L.E. , Medley G.F. & Amos W. (2011) A preliminary study of genetic factors that influence susceptibility to bovine tuberculosis in the British cattle herd. PLoS One 6, e18806. 21533277 10.1371/journal.pone.0018806 PMC3075270

[age12637-bib-0014] Falconer D.S. & Mackay T.F.C. . (1997). Introduction to Quantitative Genetics. Longman, London, UK.

[age12637-bib-0015] Fasquelle C. , Sartelet A. , Li W. *et al* (2009) Balancing selection of a frame‐shift mutation in the *MRC2* gene accounts for the outbreak of the crooked tail syndrome in Belgian Blue cattle. PLoS Genetics 5, e1000666. 19779552 10.1371/journal.pgen.1000666 PMC2739430

[age12637-bib-0016] Gammack D. , Doering C.R. & Kirschner D.E. (2004) Macrophage response to *Mycobacterium tuberculosis* infection. Journal of Mathematical Biology 48, 218–42. 14745511 10.1007/s00285-003-0232-8

[age12637-bib-0017] Gerstein M. & Zheng D. (2006) The real life of pseudogenes. Scientific American 295, 48–55. 10.1038/scientificamerican0806-48 16866288

[age12637-bib-0018] Haldane J.B.S. (1941) Selection against heterozygosis in man. Annals of Eugenics 11, 333–40.

[age12637-bib-0019] Hirotsune S. , Yoshida N. , Chen A. , Garrett L. , Sugiyama F. , Takahashi S. , Yagami K.‐I. , Wynshaw‐Boris A. & Yoshiki A. (2003) An expressed pseudogene regulates the messenger‐RNA stability of its homologous coding gene. Nature 423, 91–6. 12721631 10.1038/nature01535

[age12637-bib-0020] Hou Y. , Liu G. , Bickhart D. *et al* (2011) Genomic characteristics of cattle copy number variations. BMC Genomics 12, 127. 21345189 10.1186/1471-2164-12-127 PMC3053260

[age12637-bib-0021] Hou Y. , Liu G.E. , Bickhart D.M. , Matukumalli L.K. , Li C. , Song J. , Gasbarre L.C. , Van Tassell C.P. & Sonstegard T.S. (2012) Genomic regions showing copy number variations associate with resistance or susceptibility to gastrointestinal nematodes in Angus cattle. Functional & Integrative Genomics 12, 81–92. 21928070 10.1007/s10142-011-0252-1

[age12637-bib-0022] Houston R.D. , Haley C.S. , Hamilton A. *et al* (2010) The susceptibility of Atlantic salmon fry to freshwater infectious pancreatic necrosis is largely explained by a major QTL. Heredity (Edinbrough), 105, 318–27. 10.1038/hdy.2009.171 19935825

[age12637-bib-0023] Jørgensen C.B. , Cirera S. , Anderson S.I. , Archibald A.L. , Raudsepp T. , Chowdhary B. , Edfors‐Lilja I. , Andersson L. & Fredholm M. (2003) Linkage and comparative mapping of the locus controlling susceptibility towards *E. coli* F4ab/ac diarrhoea in pigs. Cytogenetic and Genome Research 102, 157–62. 14970696 10.1159/000075742

[age12637-bib-0024] Kandouz M. , Bier A. , Carystinos G.D. , Alaoui‐Jamali M.A. & Batist G. (2004) *Connexin43* pseudogene is expressed in tumor cells and inhibits growth. Oncogene, 23, 4763–70. 15122329 10.1038/sj.onc.1207506

[age12637-bib-0025] Koul A. , Herget T. , Klebl B. & Ullrich A. (2004) Interplay between mycobacteria and host signalling pathways. Nature Reviews Microbiology 2, 189–202. 15083155 10.1038/nrmicro840

[age12637-bib-0026] National Committee for Pig Production (2004) Annual Report 2004. National Committee for Pig Production, Copenhagen, Denmark

[age12637-bib-0027] Oldenbroek K. . (2007). Utilisation and Conservation of Farm Animal Genetic Resources. Wageningen Academic Publishers, Wageningen, the Netherlands.

[age12637-bib-0028] R Development Core Team , R (2008) r: A Language and Environment for Statistical Computing. R Foundation for Statistical Computing, Vienna, Austria.

[age12637-bib-0029] Raman K. , Bhat A.G. & Chandra N. (2010) A systems perspective of host–pathogen interactions: predicting disease outcome in tuberculosis. Molecular Biosystems 6, 516–30. 20174680 10.1039/b912129c

[age12637-bib-0030] Robertson A. (1961) Inbreeding in artificial selection programmes. Genetics Research (Cambridge) 2, 189–94.

[age12637-bib-0031] Sorokina E.M. , Feinstein S.I. , Milovanova T.N. & Fisher A.B. (2009) Identification of the amino acid sequence that targets peroxiredoxin 6 to lysosome‐like structures of lung epithelial cells. American Journal of Physiology. Lung Cellular and Molecular Physiology 297, 21. 10.1152/ajplung.00052.2009 PMC2777491 19700648

[age12637-bib-0032] Tsairidou S. , Woolliams J.A. , Allen A.R. *et al* (2014) Genomic prediction for tuberculosis resistance in dairy cattle. PLoS One, 9, e96728. 24809715 10.1371/journal.pone.0096728 PMC4014548

[age12637-bib-0033] Vanin E.F. (1985) Processed pseudogenes: characteristics and evolution. Annual Review of Genetics 19, 253–72. 10.1146/annurev.ge.19.120185.001345 3909943

[age12637-bib-0034] White M.J.D. . (1973) Animal Cytology and Evolution. Cambridge University Press, Cambridge, UK.

