# Supplementary material for: An analysis of effects of heterozygosity in dairy cattle for bovine tuberculosis resistance
Source: Anim Genet. 2018 Jan 24;49(2):103–9. doi: 10.1111/age.12637 (PMC5888165; doi:10.1111/age.12637)

Figure S1. Genotype calls scoring graph for sequencing quality control for rs43032684, where the samples are displayed in three distinct shaded areas based on their genotype calls. The three genotypic classes are represented by three distinct clusters (samples in the red region are AA genotypes, samples in the purple region are AG, and samples in the blue region are GG). Black dots represent genotypes that could not be assigned to any of the three classes.

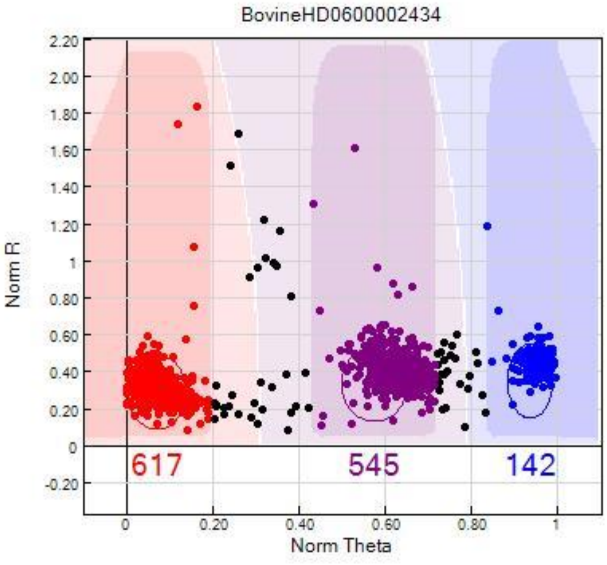

Supplement: Supplementary file 1 — Figure S1 Genotype calls scoring graph for sequencing quality control for rs43032684, where the samples are displayed in three distinct shaded areas based on their genotype calls. [file AGE-49-103-s001.pdf]
