# Supplementary material for: An analysis of effects of heterozygosity in dairy cattle for bovine tuberculosis resistance
Source: Anim Genet. 2018 Jan 24;49(2):103–9. doi: 10.1111/age.12637 (PMC5888165; doi:10.1111/age.12637)
Supplement: Supplementary file 2 — Appendix S1 Departures from Hardy‐Weinberg equilibrium. Table S1 Estimates for rs43032684 on BTA6 of the frequency of allele A (pA), and the degree of departure from Hardy‐Weinberg equilibrium (α) for cases and controls with different constraints. Figure S2 Contour plots for the cases and controls showing departures from HWE. [file AGE-49-103-s002.pdf]

Appendix S1. Departures from Hardy-Weinberg equilibrium

Departure from HWE was tested to determine the presence of heterozygote advantage/ disadvantage differentially affecting the expected genotypic frequencies in the case and control groups i.e. excess/lack of heterozygotes in the cases and controls. For the genotypic frequencies of the SNP identified by Model 2, a two-parameter model was fitted to test for departures from HWE, which may arise from non-random mating or genotype-dependent selection post conception. The genotype frequencies for, say, AA, AG and GG were modelled as: $P(AA) = p_A^2(1-\alpha) + p_A(1-p_A)\alpha$, $P(AG) = 2p_A(1-p_A)(1-\alpha)$ and $P(GG) = (1-p_A)^2(1-\alpha) + p_A(1-p_A)\alpha$, where α is the departure from non-random mating as defined by Robertson (Robertson 1965) . Denoting parameters for cases and controls with and without dashes, the maximum likelihood estimates for p_A and α and the associated values of the log-likelihood were calculated assuming: (i) $p_A = p'_A$ and $\alpha = \alpha'$; (ii) $p_A \neq p'_A$ with $\alpha = \alpha'$; (iii) $p_A = p'_A$ but $\alpha \neq \alpha'$; and (iv) the full model, $p_A \neq p'_A$ and $\alpha \neq \alpha'$. Goodness of fit was tested by likelihood ratio tests where twice the drop in log-likelihood obtained with reduced models was compared to χ^2 with degrees of freedom equal to the difference in dimensionality of the full and the reduced model.

Table S1. Estimates for rs43032684 on BTA6 of the frequency of allele A (p_A), and the degree of departure from Hardy-Weinberg equilibrium (α) for cases and controls with different constraints. The associated log-likelihoods are shown together with the likelihood ratio test statistics and degrees of freedom for comparison to χ^2. Dashed parameters denote values for cases and undashed for controls and grey text denotes constrained values. Values of $\alpha > 0$ indicate an excess of homozygotes, and < 0 a deficiency compared to Hardy-Weinberg equilibrium.

	**Cases**		**Controls**				
**Constraints**	p'_A	α'	p_A	α	**logL**	X^2	**d.f.**
$p_A = p'_A$, $\alpha = \alpha'$	0.689	0.19	0.689	0.19	-1024.59	26.769	2
$p_A \neq p'_A$, $\alpha = \alpha'$	0.658	0.10	0.724	0.10	-1019.57	16.702	1
$p_A = p'_A$, $\alpha \neq \alpha'$	0.685	-0.02	0.685	0.25	-1015.51	8.605	1
$p_A \neq p'_A$, $\alpha \neq \alpha'$	0.659	-0.02	0.722	0.24	-1011.20	-	-

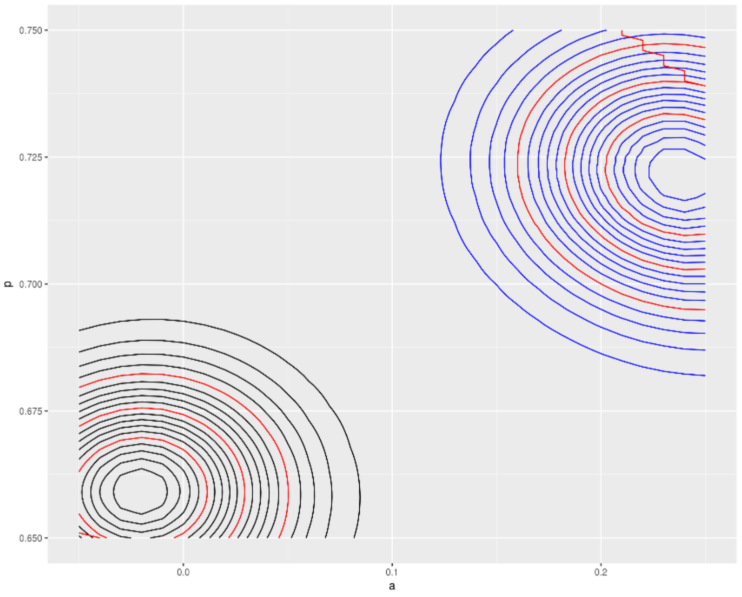

Figure S2. Contour plots for the cases and controls showing departures from HWE.

References

Robertson, A. (1965) The interpretation of genotypic ratios in domestic animal populations. *Animal science*, 3, 319-324.
