# Supplementary material for: An analysis of effects of heterozygosity in dairy cattle for bovine tuberculosis resistance
Source: Anim Genet. 2018 Jan 24;49(2):103–9. doi: 10.1111/age.12637 (PMC5888165; doi:10.1111/age.12637)
Supplement: Supplementary file 3 — Appendix S2 Standard GWAS. Table S2 Chromosome‐wide significant SNPs identified from standard GWAS and corresponding P‐values. Figure S3 Manhattan plot from standard GWAS showing significance of SNP associations based on their P‐values. Figure S4 Q‐Q plot showing observed compared to expected x2 values under the null hypothesis of no association. [file AGE-49-103-s003.pdf]

Appendix S2. Standard GWAS

Standard GWAS was conducted, using the “polygenic” and “mmscore” functions in GenABEL (R/2.15.2), and following Models (1) and (2) with 2 principal components fitted, and with x_k replacing d_k where x_k is the count of alternative alleles (0, 1 or 2) for SNP locus k. In agreement with Bermingham et al. (2014), 11 of top 12 SNPs resided on BTA13, with the top 7 SNPs in a row on BTA13, being significant at the chromosome-wide level (Table S2). Of the top 7 SNPs, 6 SNPs were those reported by Bermingham et al. (2014) (Table S2). The significance was chromosome-wide, in agreement with the significance assessment by Bermingham et al. (2014). The most significant SNP in the present study was *rs109042660* on BTA13 with -log10(P-value)=5.83 (Fig. S3). The top hit in Bermingham et al. (2014), namely rs110465273, was chromosome-wide significant also in the present study with -log10(P-value)=5.77 (Table S2). SNP *rs43705552*, also on BTA13, was chromosome-wide significant in the present study but it was not reported by Bermingham et al. (2014), while SNP *rs109809949* detected by Bermingham et al (2014) was marginally not significant in the present study (-log10(P-value)=5.45). These results demonstrate the robustness of the outcomes despite using different statistical association methods and models from Bermingham et al. (2014). Small differences were expected due to slight modifications in procedures and models.

Table S2. Chromosome-wide significant SNPs identified from standard GWAS and corresponding P-values. In bold are shown the SNPs reported in Bermingham et al. (2014).

SNP		Chromosome	$-\log_{10}$(P-value)
**BovineHD1300020589**	**rs109042660**	**13**	**5.83**
**BovineHD1300020586**	**rs42494342**	**13**	**5.80**
**BovineHD1300020584**	**rs42494357**	**13**	**5.77**
**BovineHD1300020585**	**rs110465273**	**13**	**5.77**
**BovineHD1300020590**	**rs137562332**	**13**	**5.77**
**BovineHD1300020591**	**rs132841890**	**13**	**5.75**
BovineHD4100010384	rs43705552	13	5.62
**Genome-wide threshold**			7.06
**Chromosome-wide threshold**			5.49

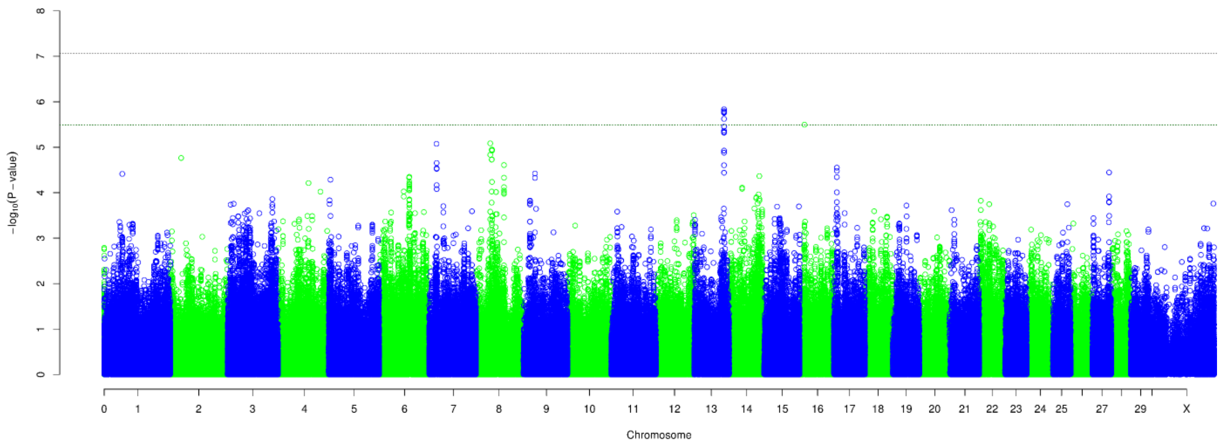

Figure S3. Manhattan plot from standard GWAS showing significance of SNP associations based on their P-values. The green line represents the chromosome-wide threshold for BTA13, and the black line represents the genome-wide threshold.

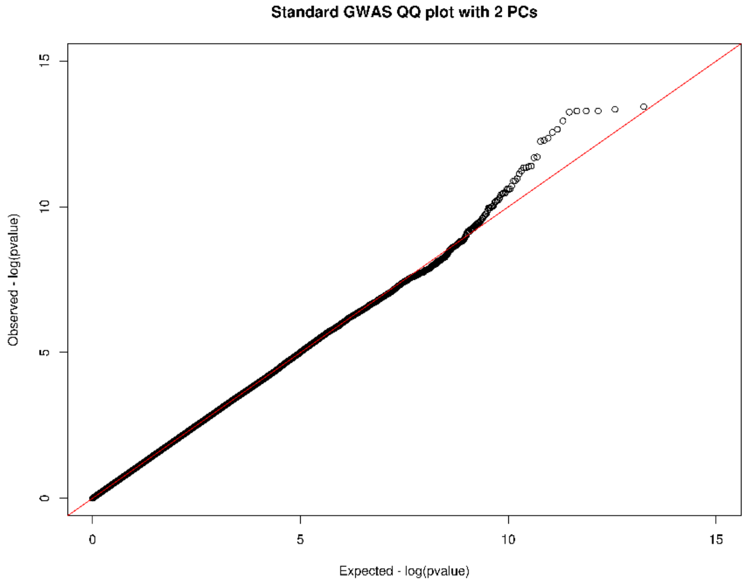

Figure S4. Q-Q plot showing observed compared to expected x^2 values under the null hypothesis of no association.
